# Viral Dose and Immunosuppression Modulate the Progression of Acute BVDV-1 Infection in Calves: Evidence of Long Term Persistence after Intra-Nasal Infection

**DOI:** 10.1371/journal.pone.0124689

**Published:** 2015-05-08

**Authors:** Rebecca Strong, Severina Anna La Rocca, David Paton, Emmanuelle Bensaude, Torstein Sandvik, Leanne Davis, Jane Turner, Trevor Drew, Rudiger Raue, Ilse Vangeel, Falko Steinbach

**Affiliations:** 1 Virology Department, Animal Health and Veterinary Laboratories Agency (AHVLA), Addlestone, United Kingdom; 2 Veterinary Medicine Research & Development, Zoetis, Belgium; CEA, FRANCE

## Abstract

Bovine viral diarrhoea virus (BVDV) infection of cattle causes a diverse range of clinical outcomes from being asymptomatic, or a transient mild disease, to producing severe cases of acute disease leading to death. Four groups of calves were challenged with a type 1 BVDV strain, originating from a severe outbreak of BVDV in England, to study the effect of viral dose and immunosuppression on the viral replication and transmission of BVDV. Three groups received increasing amounts of virus: Group A received 10^2.55^TCID_50_/ml, group B 10^5.25^TCID_50_/ml and group C 10^6.7^TCID _50_/ml. A fourth group (D) was inoculated with a medium dose (10^5.25^TCID_50_/ml) and concomitantly treated with dexamethasone (DMS) to assess the effects of chemically induced immunosuppression. Naïve calves were added as sentinel animals to assess virus transmission. The outcome of infection was dose dependent with animals given a higher dose developing severe disease and more pronounced viral replication. Despite virus being shed by the low-dose infection group, BVD was not transmitted to sentinel calves. Administration of dexamethasone (DMS) resulted in more severe clinical signs, prolonged viraemia and virus shedding. Using PCR techniques, viral RNA was detected in blood, several weeks after the limit of infectious virus recovery. Finally, a recently developed strand-specific RT-PCR detected negative strand viral RNA, indicative of actively replicating virus, in blood samples from convalescent animals, as late as 85 days post inoculation. This detection of long term replicating virus may indicate the way in which the virus persists and/or is reintroduced within herds.

## Introduction

Bovine viral diarrhoea virus (BVDV) is a major pathogen of cattle with worldwide economic impact [1]. It is a member of the *Flaviviridae* family, genus *Pestivirus*, which also includes border disease virus and classical swine fever virus. There are two recognised species of BVDV (-1 and -2) that can be further subdivided into genotypes [2] and a third related virus tentatively referred to as BVDV-3 [3]. Transmission of BVDV is generally considered to be via droplet spread between contact animals. The virus replicates in the mucosal epithelium and associated lymphoid tissues, and this is followed by viraemia and subsequent viral replication in different organ systems throughout the body [4–7]. Infectious virus can be recovered from serum between 4 and 15 days post infection. Specific neutralizing antibodies appear at a low level from 10–14 days post infection, but their titre continues to increase slowly for 8–10 weeks. Both early immunosuppression and subsequent persistence of BVDV antigens in lymphoid tissues contribute to this [8].

Postnatal infections with BVDV generally remain subclinical, even though a transient leukopenia may occur. With more virulent strains, infections can be associated with a wide range of clinical signs including fever, diarrhoea, as well as respiratory and/or reproductive dysfunctions. The latter may be caused by the immunosuppressive effect of BVDV and secondary infections rather than the virus itself [9]. Fatal cases of acute BVD caused by virulent strains have also been described [10–11]. Overall, the pathogenesis of BVDV is influenced by poorly defined factors beyond viral strain virulence such as host animal age and immunological status, the latter being affected by stress or co-infection by other pathogens [12–14].

In this study, the effects of viral dose and simulated immunological stress were studied in groups of calves inoculated with a strain of BVDV-1 that was known to have the potential to cause overt disease. The study also provided an opportunity to compare different methods to detect infectious virus and viral RNA. Our study demonstrates not only an influence of viral dose and immunosuppression on the replication of BVDV-1, but also that BVDV is replicating long after the clinical course of the disease is over.

## Materials and Methods

### Viruses

The non-cytopathic BVDV-1 strain Ho916 was originally isolated from a clinically severe outbreak of BVDV associated with a number of deaths in cattle in southern England [10]. This isolate was harvested from foetal bovine turbinate (fBT) cells and used to prepare inocula of low, medium and high doses containing 10^2.55^, 10^5.25^ and 10^6.7^ 50% tissue culture infectious doses (TCID_50_) of virus in volumes of 2-10ml respectively.

### Study Design

Approximately 3–4 months of age calves were randomly allocated to groups of 12 or 13 calves or were introduced as sentinel animals. Prior to inoculation, calves tested negative for BVDV antibodies by a BVD in-house ELISA (AHVLA). In group A, calves were inoculated intranasally with a low dose (10^2.55^ TCID_50_) Ho916 and two BVDV naïve calves were introduced 2 days post infection (dpi) as sentinels, to assess for potential horizontal transmission of viral infection. Pairs of calves were euthanised for post mortem examination at various dpi, with the remaining calves’ euthanised 42 dpi. In group B, 12 calves were inoculated with a medium dose (10^5.25^ TCID_50_) Ho916. Two naïve calves were introduced at 4 dpi as sentinels. Sentinel animals were removed 35 dpi and euthanised 49 dpi. All inoculated animals were euthanised for post mortem by 80 dpi. In group D, 13 animals were inoculated in the same manner as group B animals, but also received 10mg dexamethasone (DMS) intramuscularly on the day of inoculation, and for the following 4 days. The administration of DMS was intended to simulate a state of immunosuppression in inoculated calves [15]. Sentinels were removed 36 dpi and euthanised at 57 dpi. A further two calves were introduced as sentinels at 50dpi and euthanised at 80 dpi. All inoculated calves were euthanised by 80 dpi for post mortem. Clinical examination of the calves was performed on a daily basis by a veterinarian and clinical status was recorded according to a clinical scoring system (S1 Table). Rectal temperatures were measured daily and animals were considered to be exhibiting a fever when the rectal temperatures were above 39.5°C [16]. Nasal swab and blood samples, both clotted and anti-coagulated with EDTA, were collected regularly, including every second day during the early course of the experiments.

Subsequently, another trial was carried out to test the results and hypothesis derived from the first part of the study using high doses of BVDV-1 strain Ho916 (group C, 10^6.7^ TCID_50_) inoculated to eight 7 week old and five 5 month old calves. Using two age groups could further indicate the importance of the immune system for the outcome of BVDV infection, which is maturing during the first year of age. The animals were carefully selected as before and the housing was comparable (indoors) in both trials and can be referred to as high health management facilities. The acute phase of the disease was carefully monitored (but due to ethical considerations without introducing sentinel animals in this trial), while focusing on comparing disease progression in different age animals. Seven week old calves were euthanised 21 dpi, whilst 5 month old calves were euthanised for post mortem 77 and 85 dpi.

### Ethics statement

The initial study (Group A, B and D) was carried out at AHVLA laboratories in compliance with the Animal (Scientific Procedures) Act 1986 and carried out in accordance to Home office project licence 90/00886. The later study was (Group C) was conducted at Charles River Laboratories under Home Office License Number PPL 60/3035 (Procedure A12) and approved by the Pfizer Animal Health Animal Welfare Committee.

### Virus isolation

Sera, white blood cells (WBCs) and nasal swabs were tested for infectious BVDV in microplate BT cell cultures which were in routine use for field diagnosis and quality controlled for sensitivity of virus isolation (VI). After a four day incubation period, culture plates were fixed and immunostained for BVDV antigen [17]. Sera were diluted 1:10 in cell culture medium to a final volume of 100μl. WBCs were separated from whole EDTA blood by removing the buffy coat, using 0.9% ammonium chloride to lyse red blood cells, followed by centrifugation at 3,000g for 10 minutes. After resuspension in ammonium chloride and recentrifugation, the WBC pellet was resuspended in PBS and then frozen at—70°C. Nasal swab tips were cut off into bijoux containing 0.5 ml of culture fluid, and the resulting eluates diluted 1:5 before use.

### RT-PCR

EDTA blood, serum and nasal swabs were also tested for the presence of viral nucleic acid, using a closed one-tube RT-nested PCR with a pestivirus-specific TaqMan fluorescent probe [18]. Briefly, total RNA was isolated using the TRIzol method (Life Technologies) according to manufacturer’s recommendation. Total RNA was dissolved in 20μl of water and a 3μl aliquot used as template for the RT-PCR reaction. For selected whole blood samples, RNA was extracted from 140μl of blood using the QIAamp viral RNA extraction kit (Qiagen) according to the manufacturer’s procedure. Viral RNA was detected using a short target real-time PCR assay as previously described [19].

### Negative strand RT-PCR

A negative strand specific RT-PCR was developed to detect the presence of the replicative intermediate evident during BVDV genome replication [20]. Briefly cDNA was produced using the Thermoscript (Invitrogen) RT components. Reverse transcription was performed using 6μl of RNA with 0.3μl of the 106-TAG primer (5’- tcatggtggcgaataaccatrcccktagtaggactagc-3’), 1μl of dNTPs and 4.7μl of dH_2_0. The sample mix was heated at 95°C for 2 min and placed on ice for 2 mins. 4μl of 5X cDNA synthesis buffer, 1μl DTT (0.1M), 1μl of RNAse OUT, 0.25μl Thermoscript enzyme and 1.75μl of dH_2_0 were combined and added to the samples. Reverse transcription was performed at 58°C for 60 mins, followed by 5 mins at 85°C. Control RT were performed using serial dilutions of a 77-mer synthetic negative strand RNA, starting at a dilution of 1x10^7^ molecules/μl. lμl of cDNA was combined with 12.5μl of Brilliant Blue master mix (Stratagene), 1μl of 190R primer (10μM, 5’- gygtcgaaccaytgacgact-3’), 1 μl of TAG primer (10μM, 5’-tcatggtggcgaataa-3’), 0.5μl of P-162 probe (5μM, 5’-FAM- tggatggcykaabccctgagtacag-EDQ-3’) and 6μl of dH_2_0. The PCR was performed with the following parameters: 50°C 10 min, 95°C 2 min, and 50 cycles of 95°C for 15 sec and 60°C for 30 sec. A serial dilution of a synthetic positive strand RNA, starting at 1 x 10^7^ molecules/ul was used to test for strand specificity and the synthetic RNAs with no primer and no RT were tested for any evidence of self-priming. This assay is able to detect negative strand synthetic RNA at 1 x 10^3^ molecules/ul.

### Serum neutralisation tests

Blood samples without anti-coagulant were collected on day 0 (prior to inoculation), and every other day until 21 dpi and weekly thereafter. Samples were only collected on day 0 and 21dpi for group C calves. Antibodies against BVDV were titrated using a microtitre serum virus neutralisation test using BVDV strain Oregon C24V as a positive control at 100 TCID_50_. Titres greater than or equal to 1 in 10 were considered positive [17].

### Haematology

EDTA blood samples for WBC, platelet and monocyte counts were collected five and three days before, and on the day of inoculation, to form a baseline. Samples were taken every other day until 21 dpi, then weekly until the end of the experiment. For group C animals, EDTA blood samples were taken daily from 2 days prior to inoculation to 18 dpi. Counts were performed using an automated analyser either on the day of collection or after two days of storage at 4°C.

### Statistical analysis

Results were expressed as mean values ± SEM. Statistical significance between individual animals from each group with common dpi and overall group means were determined using a one way ANOVA test. Statistical significance was considered when p < 0.05.

## Results

**Fig 1:**
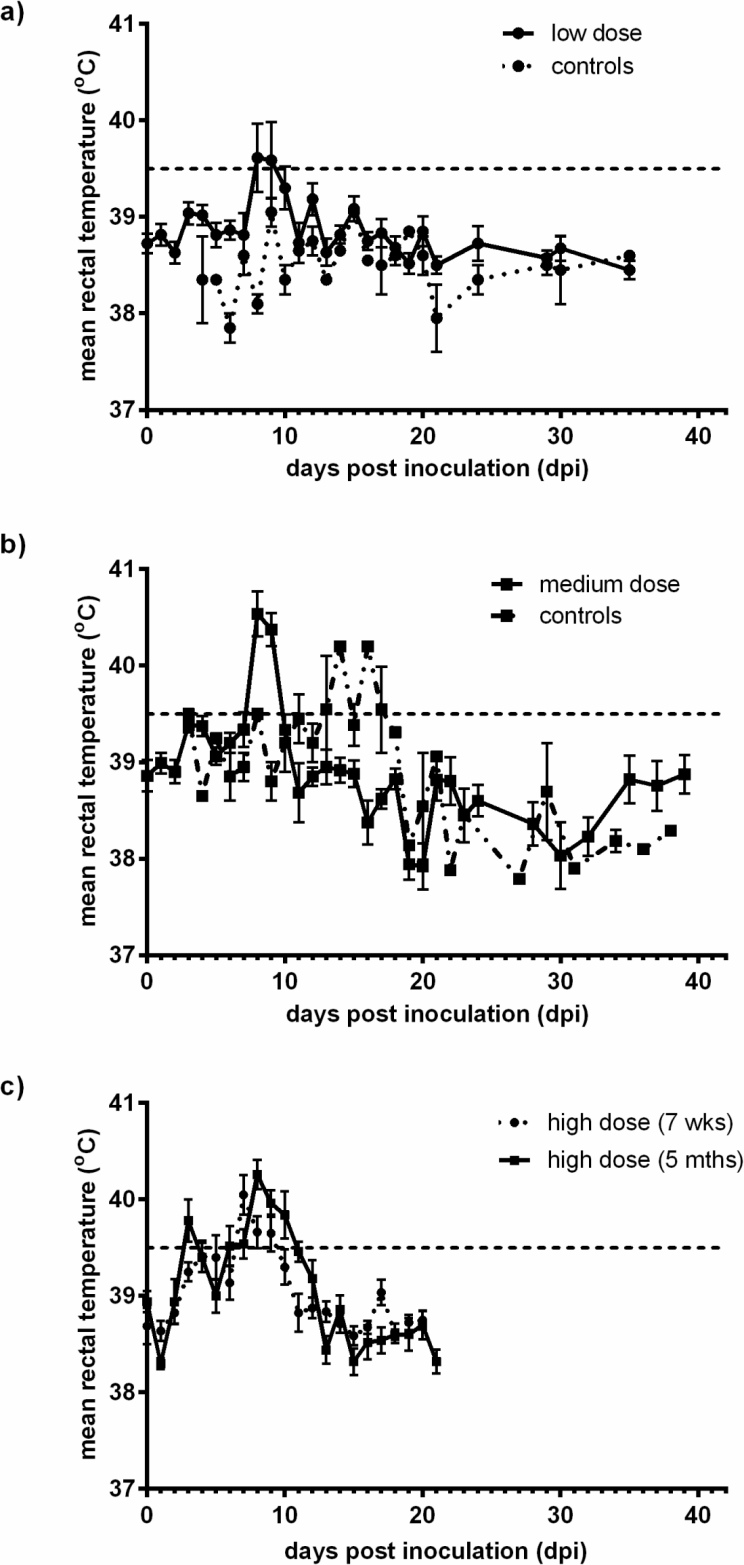
Mean rectal temperatures during the acute stage of infection with low, medium, and high dose BVDV-1. Mean rectal temperatures ± SEM were plotted for calves inoculated with Ho916 a) Group A—low dose, 10^2.55^ TCID_50_, b) Group B—medium dose b—10^5.25^ TCID_50_ and c) Group C—high dose, 10^6.7^ TCID_50_, and for the sentinel animals in group A and B. Calves inoculated with a low dose were euthanised at 35 dpi. For the medium dose calves, only the acute phase of infection is shown, up to 38 dpi and 39 dpi for sentinel and infected calves respectively. In group C, the 7 week old calves were euthanised at 21 dpi, and for the 5 month old calves, only data from the acute phase of the infection (up to 21 dpi) is shown.

In all groups, disease progression followed a predicted pattern and the severity of clinical signs clearly correlated with the infectious dose. In group A (low dose BVDV-1) only four out of eight animals were febrile at 8 dpi (Fig 1) with no other distinct clinical signs. Importantly, the sentinel animals did not become infected, as indicated by a lack of seroconversion. The group B calves (medium dose BVDV-1) exhibited mild depression, moderate diarrhoea, inappetence and a cough at a peak around 12 dpi, as well as a pyrexia between 8 and 9 dpi. The sentinel animals introduced to group B showed mild diarrhoea and a lack of appetite. One of the two sentinel animal introduced became febrile by 12 dpi and seroconverted at 30 dpi. The 7 week old group C calves showed a range of clinical signs including coughing, diarrhoea, dyspnoea, depression, inappetence and a fever by 7 dpi which lasted for 3 days. The 5 month old group C calves showed fewer clinical signs compared to the 7 week old calves, including cough and nasal discharge, and exhibited a fever biphasically with peaks at 3 dpi, and 6 dpi, lasting until 10 dpi. Overall the group C calves showed an increased range of clinical signs with a prolonged fever compared to groups A and B.

**Fig 2:**
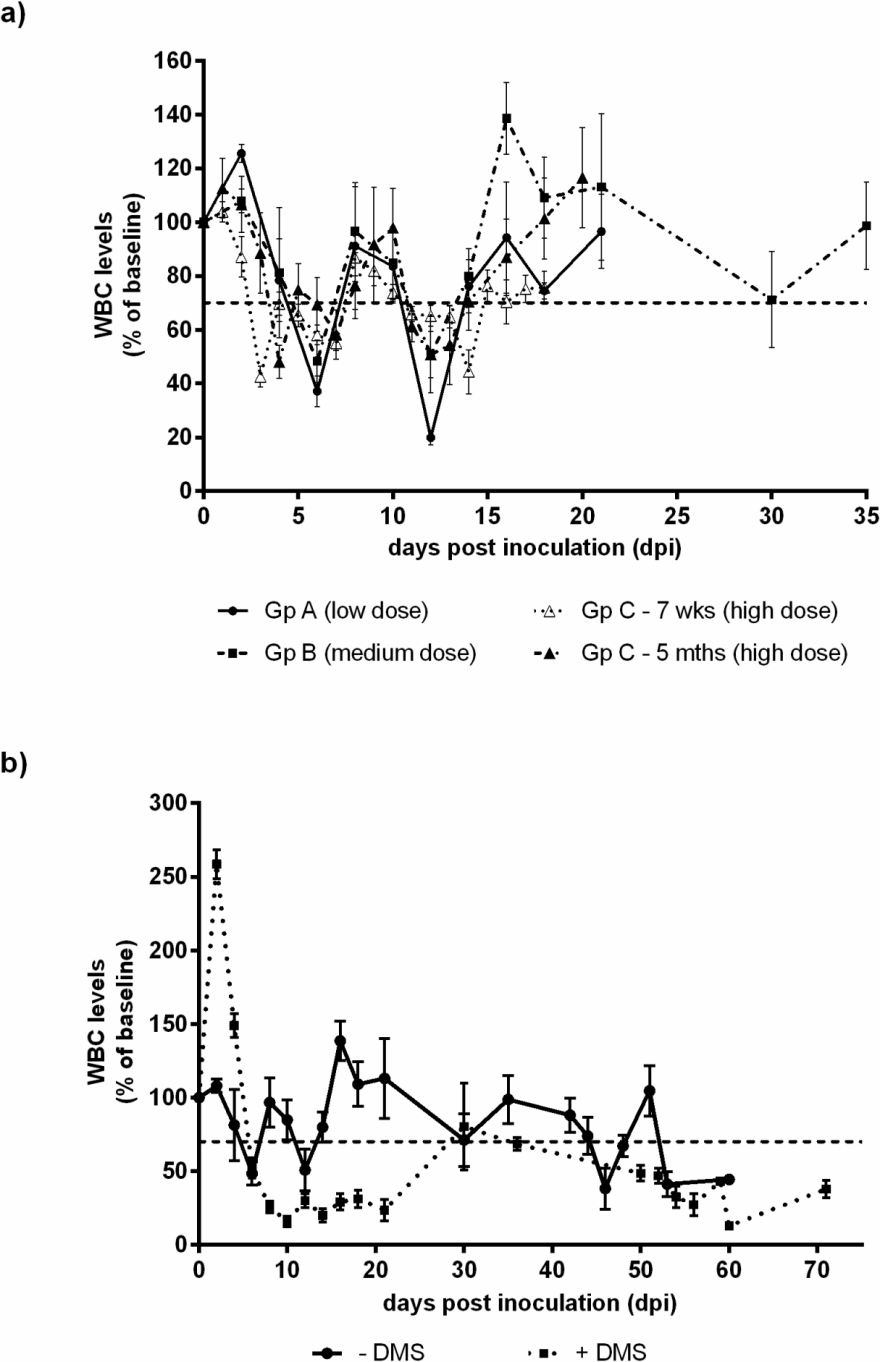
Mean WBCs counts during the acute stage of infection. a) Mean WBCs ± SEM were plotted as a percentage of baseline counts in group A (low dose inoculum; only those animals that exhibited fever and/or positive by either RT-PCR or virus isolation were included e.g. 7 out of 12 calves) and group B (medium dose inoculum). WBC counts are shown for the acute phase of infection in group C (high dose inoculum). b) Mean WBCs ± SEM were plotted as percentages of baseline counts over the duration of the study, namely 60 dpi for group B (medium dose) and 71 dpi for group D (medium dose with DMS administered at time of inoculation).

White blood cell (WBC) and platelet counts were determined for all groups, with the exception of group C where no platelet counts were recorded. WBC and platelet levels were plotted as percentages of the pre-inoculation baseline (Fig 2). Calves were regarded as leukopaenic when the WBC counts dropped below 70% of the baseline level. In all groups, a biphasic leukopenia was observed with an earlier drop in the high dose group compared to either the medium or low dose group. Interestingly, the 7 week old animals became leukopaenic before the 5 month old calves. In all groups, WBC counts returned to above the 70% of the baseline level by 15 dpi. Thrombocytopaenia was observed in both groups A and B, with a slower recovery of platelet levels in group B (medium dose) compared to group A (low dose) (S1 Fig).

In all three groups, a proportion of the inoculated animals tested positive by either virus isolation or RT-PCR independent of the sample types (Table 1). As expected, virus isolation was less sensitive than RT-PCR, and detected virus for a shorter period. In group A, 50% of inoculated animals tested virus isolation and/or RT-PCR positive during the study, whilst sentinel animals remained negative. Over the course of the study, all group B animals tested positive by either RT-PCR and/or virus isolation for each sample type taken from 4 dpi, but not every animal tested positive on each day. In group B, viral RNA was present in serum samples and nasal swabs up to 30 and 35 dpi, respectively, compared to 16 and 21 dpi for group A. The serum sample from one sentinel animal, in group B, that had exhibited a fever, tested positive by both RT-PCR and virus isolation. In group C, virus was isolated from both the 7 week old and 5 month old animals from both WBC and nasal swab samples, as early as 1 dpi for nasal swab samples. During the acute stage of infection, a higher proportion of group C animals tested positive on each sampling day, compared to either group A or B. Virus isolations from WBCs of 7 week old calves in group C tested positive from 3 to 14 dpi, with all animals testing positive 4 to 6 dpi and 8 dpi. For the 5 month old calves, virus was isolated from 2 to 10 dpi, with all animals testing positive 5, 6 and 8 dpi. Maximum titres reached 10^3.55^ TCID_50_/ml on day 6.

**Table 1:** Summary of virological findings for increasing dose of Ho916 during acute infection and for treatment with and without DMS.

Group	Inoculum	No of animals (Day of introduction/ removal)	Sample	Assay	Range of detection (days)	Peak detection (days)
			EDTA blood	RT-PCR	4–30	-
			Serum	RT-PCR	4–16	-
A	10^2.55^	12		VI	5–10	-
			Nasal swab	RT-PCR	4–21	-
				VI	6–10	-
	Sentinels	2	Serum	RT-PCR	0	-
		(2dpi—30dpi)		VI	0	-
			EDTA blood	RT-PCR	4–80	4–14,18
			Serum	RT-PCR	4–47	8
B	10^5.5^	12		VI	4–8	-
			Nasal swab	RT-PCR	2–60	4–8
				VI	4–10	-
	Sentinels	2	Serum	RT-PCR	14–16	-
		(4 dpi—35 dpi)		VI	14	-
			WBC (7 wk)	VI	3–14	4–6, 8
		8 (7wk)	WBC (5 mth)	VI	2–10	5–6, 8
C	10^6.7^					
		5 (5 mth)	Nasal swab (7 wk)	VI	1–15	4, 6–9, 11–12
			Nasal swab (5 mth)	VI	1–13	2, 4, 7–10
			EDTA blood	RT-PCR	4–80	4–21, 36–80
			Serum	RT-PCR	4–60	6–14
D	10^5.5^	13		VI	4–18	6–10
	+ DMS					
			Nasal swab	RT-PCR	2–80	8–21, 36, 71
				VI	4–21	8–14
		2	Serum	RT-PCR	12–21	14–18
		(4dpi—36 dpi)		VI	12–16	14–16
	Sentinels					
		2	Serum	RT-PCR	0	0
		(50dpi—80 dpi)		VI	0	0

Group A animals were all euthanased by 42 dpi

Peak detection indicates the days on which all animals within the group were positive for the assay.

WBC = White blood cells, VI = Virus isolation, 7 wk = Seven weeks old calves, 5 mth = Five months old calves

In group A, those animals with detectable virus replication seroconverted at 14 dpi. One further animal seroconverted at 30 dpi, even though no viral RNA or virus was detected in any of the samples taken from this animal throughout the study. In group B, 5 out of 6 calves alive at 14 dpi had seroconverted, and the remaining animal seroconverted at 16 dpi. The sentinel animal that tested positive by virus isolation and RT-PCR seroconverted at 30 dpi. For group C, all calves seroconverted within 21 dpi, and the 7 week old animals displayed a higher mean titre compared to the 5 month old calves (S2 Fig).

Dexamethasone (DMS) was administered to a group of calves to artificially simulate immunosuppression alongside a BVDV-1 infection (group D). These calves were inoculated with a medium dose of Ho916, as done for group B, with the addition of DMS treatment at the time of inoculation and four days following. Clinical effects were more severe in group D than group B. One animal from group D had to be euthanised on welfare grounds at 32 dpi. The administration of DMS caused an increase in WBC levels at first, followed by a significant decrease (Fig 2B). Moreover, WBC levels following DMS treatment showed a statistically significant decrease (p<0.01) compared to group B, which had no DMS treatment. Thrombocytopenia was also observed during the acute infection. In group D, a marked reduction in platelet counts was observed (29% of the pre-inoculation level) compared to the drop in platelet levels observed in group B (37% of the pre-inoculation level) (S3 Fig).

The administration of DMS at the time of BVDV inoculation prolonged the period over which virus was detected (Table 1). Serum samples from group D, were still positive by VI at 18 dpi compared to 8 dpi in group B and similarly the nasal swabs were still positive at 21 dpi in group D compared to 10 dpi in group B. The presence of viral RNA in both serum samples and nasal swabs was also prolonged in group D, and there was a higher number of peak detection days in comparison to group B, suggesting that immunosuppression enhance the ability of BVDV to establish a productive infection. In group D, both sentinel animals tested positive by virus isolation and RT-PCR over the range 12 to 21 dpi whilst only one of the two sentinel animals in group B had tested positive and on fewer days. Therefore, alongside an increased shedding, immunosuppression also increased the transmission of virus to sentinel calves. In contrast, the administration of DMS at the time of inoculation slowed the rate at which neutralising antibodies were produced. In group D, two out of the seven calves alive at 14 dpi were seropositive and the remaining animals had seroconverted by 30 dpi. The two sentinel animals that tested positive by virus isolation and RT-PCR seroconverted by 30 dpi, similar to the group B sentinel animal. The sentinel calves introduced at 50dpi, after the acute infection had been cleared, showed no signs of infection and were negative by VI and RT-PCR in all samples taken.

In group B, viral RNA was detected at 80 dpi, long after the animals had recovered from the acute stage of infection. In light of this, the 5 month old calves from group C were maintained until 85 dpi and EDTA blood samples were collected at regular intervals until the end of the study. The samples were tested both by a diagnostic short target PCR [19], and by a second RT-PCR delineated from this, which specifically detects negative strand RNA [20]. Not only could viral RNA be detected until 85 dpi but it was also possible to detect negative strand viral RNA until the end of the study, indicating a continued active replication of BVDV-1 beyond acute infection (Table 2).

**Table 2:** Ct values for short target and negative strand RT-PCR.

Virus	PCR	Days post inoculation				
		43	57	71	80	85
BVDV-1	Short target	40.39	38.70	36.81	38.12	39.87
	Negative strand	40.48	40.87	40.18	38.56	40.84

## Discussion

The primary purpose of this study was to better understand the effect of dose on BVDV-1 infection and the influence of immunological parameters on clinical disease. In all treatment groups, disease progressed as described in previous studies, with transient pyrexia associated with other clinical signs, transient leukopaenia and thrombocytopaenia [21–24]. Also, clinical findings for Group B calves (10^5.55^ TCID_50_) were reproducible in a subsequent experimental study based on the model established here, with Ho916 administered at dose of 10^5^ TCID_50_ [25]. Also Ridpath et al. (2007) reported that there was little difference in clinical progression between calves inoculated with three different doses covering two logs (10^4^ to 10^6^ TCID_50_) [26]. However this study provides data from calves inoculated with a wider range of dose covering almost 4 logs (10^2.55^ to 10^6.7^) and shows that dose indeed has an effect on the range of symptoms and the severity of disease. Many experiments have been reported which involve the inoculation of calves with BVDV in doses exceeding 10^5^ TCID_50_ [27–29], but no data seemed to exist on inoculation with low doses of BVDV-1 as described here (10^2.55^ TCID_50)_. As there is clearly a high variability in the virulence of different BVDV strains, information is lacking about what might constitute a minimum infective dose for a calf, also described as a Calf infectious dose 50% (CID_50_). Although this was not intended in the experimental design, the low dose viral inoculum seemed to equate to approximately one CID_50_ for Ho916, since only three out of six calves remaining at 14dpi had seroconverted. In comparison to the calves, which received higher doses, group A animals showed less clinical signs and no post challenge viral transmission. However, the duration of viral isolation from blood and the onset of seroconversion were no different from the other groups. The effect of age and thus of immunological maturity on the progression of clinical disease was also apparent. The 7 week old calves exhibited a wider range of clinical signs, were slower at clearing the virus, and delayed in recovering normal WBC counts compared to 5 month old calves. Similarly, Hamers et al. (2003) reported more pronounced clinical symptoms in younger animals [30].

The short period during which virus could be isolated from group A, B and C calves, and the distinct but non-fatal clinical signs observed are typical of acute infection with moderately virulent viruses [31]. The results presented here are consistent with previous observations that BVDV infections tend to be less severe under experimental circumstances than in the field [12, 32, 33] and indeed Larska et al. (2012) confirmed that infection with Ho916 was less pathogenic under experimental conditions [25]. This highlights the potential importance of host and environmental factors in the clinical outcome of the disease.

Group D calves treated with DMS at the time of inoculation showed more severe clinical signs. This finding confirms that of other research groups, where calves treated with DMS developed lethal infections after being given large doses of BVDV intravenously [33–34]. DMS is known to interfere with both the innate and the adaptive immune responses [15] crucial to the initial control of infections in naïve animals. Another striking difference in group D animals was the prolonged viraemia and nasal shedding of virus. This is also explained by a delayed immune response, due to the chemically-induced immunosuppression. Altogether, the evidence suggests that the episodes of severe BVD seen occasionally in the field may be due to immunosuppression. Thus, while BVDV itself causes immunosuppression, the clinical severity of BVD is exacerbated by immunosuppression caused by other agents.

Viral RNA was found in EDTA blood samples in group B and D up to 80 dpi and group C up to 85dpi. Together with other reports, this illustrates the ability of BVDV to remain “hidden” long after the initial clinical signs have disappeared and the animals have seroconverted [7, 28, 35–37]. The ability to detect viral RNA in blood long after the initial infection is of diagnostic relevance. The long term infection demonstrated here highlights an issue of clinical, pathological and virological nomenclature. While acute and chronic viral infections (other than BVDV) are often associated with clinical signs being present or measurable, latent or persistent infections are more generally associated with an absence of clinical signs. The demonstration of RNA replicative intermediate as late as 85 dpi shows that the virus is still replicating long after the end of the clinical course, and the infection therefore transgressed into a persistent form compared to a latent one where no replication would be detectable. It remains unresolved at present in which cells the virus replicates since complex sorting procedures dissecting at least the major myeloid and lymphoid cell populations were not possible to conduct here. Also as virus isolation was impossible here and elsewhere [28] the possibility of a persistent, but restricted infection, where the production of virions is suppressed, has to be considered as a subject of future studies.

The term persistent infection for BVDV has so far exclusively been reserved for animals infected *in utero* which shed virus throughout their lives and thus represent the biggest risk of BVDV dissemination. Despite the continuous presence of BVDV RNA in the blood, and in some cases in the nasal swabs of group D calves until 80 dpi, no horizontal transmission occurred after the clinical course of the disease, which is consistent with a previous report by Collins et al. (2009)[28]. Previous studies have shown the ability of naïve calves to become infected after inoculation of blood or semen from recovered animals [28, 38]. The persistence of viral replication in cells, after the appearance of antibody and in the absence of detectable infectious virus, is suggestive of an impaired cellular immunity, associated with the incomplete removal of infected cells. The fact that the prolonged RNAaemia is associated with the higher dose inoculation seems to indicate that persistence is indeed caused by the virus itself, rather than by external factors.

Together, these findings have important implications for our understanding of the pathogenesis, immunity and transmission of BVDV, as well as for the use of animal models in vaccine and cross-protection studies. However, more studies are required to ascertain how long BVDV may persist in its host, and to establish the form and cellular location in which the virus persists after acute infection.

## Supporting Information

S1 Fig Mean platelet counts. Mean platelet counts ± SEM were plotted as a percentage of baseline counts in group A (low dose inoculum; only those animals that exhibited fever and/or positive by either RT-PCR or virus isolation were included e.g. 7 out of 12 calves) and group B (medium dose inoculum). WBC counts are shown for the acute phase of infection in group C (high dose inoculum).
(PPT)

S2 Fig Mean platelet counts. Mean platelet counts ± SEM were plotted as percentages of baseline counts over the duration of the study, namely 60 dpi for group B (medium dose) and 71 dpi for group D (medium dose with DMS administered at time of inoculation).
(PPT)

S3 Fig Serological response. Mean serum neutralising antibody titres (log_2_ transformed) ± SEM are plotted for group C (high dose inoculum) for the 7 weeks old and 5 month old animals.
(PPT)

S1 Table Clinical Scoring System. (DOC)

## References

[1] Houe H. Epidemiological features and economical importance of bovine virus diarrhoea virus (BVDV) infections. Vet Microbiol. 1999; 64; 89–107. 10028165 10.1016/s0378-1135(98)00262-4

[2] Thiel HJ, Collett MS, Gould EA, Heinz FX, Meyers G, Purcell RH, et al Family Flaviviridae In: Fauquet CM, Mayo MA, Maniloff J, Desselberger U, Ball LA, editors. Virus Taxonomy—Eighth Report of the International Committee on Taxonomy of Viruses. San Diego: Academic Press; 2005 pp. 981–998.

[3] Schirrmeier H, Strebelow G, Depner K, Hoffmann B, Beer, M. Genetic and antigenic characterization of an atypical pestivirus isolate, a putative member of a novel pestivirus species. J Gen Virol. 2004; 85; 3647–3652. 15557237 10.1099/vir.0.80238-0

[4] Bielefeldt Ohmann H. Pathogenesis of bovine viral diarrhoea-mucosal disease: distribution and significance of BVDV antigen in diseased calves. Res Vet Sci. 1983; 34; 5–10. 6300980

[5] Marshall DJ, Moxley RA, Kelling CL. Distribution of virus and viral antigen in specific pathogen-free calves following inoculation with noncytopathic bovine viral diarrhea virus. Vet Pathol. 1996; 33; 311–318. 8740705 10.1177/030098589603300308

[6] Bruschke CJ, Weerdmeester K, Van Oirschot JT, Van Rijn PA. Distribution of bovine virus diarrhoea virus in tissues and white blood cells of cattle during acute infection. Vet Microbiol. 1998; 64; 23–32. 9874100 10.1016/s0378-1135(98)00249-1

[7] Givens MD, Riddell KP, Edmondson MA, Walz PH, Gard JA, Zhang Y, et al Epidemiology of prolonged testicular infections with bovine viral diarrhea virus. Vet Microbiol. 2009; 139; 42–51. 10.1016/j.vetmic.2009.04.029 19473788

[8] Brownlie J. The pathogenesis of bovine virus diarrhoea virus infections. Rev Sci Tech. 1990; 9; 43–59. 2132154 10.20506/rst.9.1.491

[9] Baker JC. The clinical manifestations of bovine viral diarrhea infection. Vet Clin North Am Food Anim Pract. 1995; 11; 425–445. 8581856 10.1016/s0749-0720(15)30460-6

[10] Hibberd RC, Turkington A, Brownlie J. Fatal bovine viral diarrhoea virus infection of adult cattle. Vet Rec. 1993; 132; 227 8451822 10.1136/vr.132.9.227

[11] Pellerin C, van den Hurk J, Lecomte J, Tussen P. Identification of a new group of bovine viral diarrhea virus strains associated with severe outbreaks and high mortalities. Virology. 1994; 203; 260–268. 8053150 10.1006/viro.1994.1483

[12] Bolin SR, Ridpath JF. Differences in virulence between two noncytopathic bovine viral diarrhea viruses in calves. Am J Vet Res. 1992; 53; 2157–2163. 1334641

[13] David GP, Crawshaw TR, Gunning RF, Hibberd RC, Lloyd GM, Marsh PR. Severe disease in adult dairy cattle in three UK dairy herds associated with BVD virus infection. Vet Rec. 1994; 134; 468–472. 8059512 10.1136/vr.134.18.468

[14] Ridpath J. The contribution of infections with bovine viral diarrhea viruses to bovine respiratory disease. Vet Clin North Am Food Anim Pract. 2010; 26; 335–348. 10.1016/j.cvfa.2010.04.003 20619188

[15] Munck A, Guyre PM. Glucocorticoid physiology, pharmacology and stress. Adv Exp Med Biol. 1986; 196; 81–96. 3012985 10.1007/978-1-4684-5101-6_6

[16] Hofmann W, Bostedt H, Weitze KF, Stanek C. Der klinische Untersuchungsgang. Rinderkrankheiten, Bd.1, Innere und chirurgische Erkrankungen. 2005.

[17] Edwards S. The diagnosis of bovine virus diarrhoea-mucosal disease in cattle. Rev Sci Tech. 1990; 9; 115–130. 1966717 10.20506/rst.9.1.486

[18] McGoldrick A, Bensaude E, Ibata G, Sharp G, Paton DJ. Closed one-tube reverse transcription nested polymerase chain reaction for the detection of pestiviral RNA with fluorescent probes. J Virol Methods. 1999; 79; 85–95. 10328538 10.1016/s0166-0934(99)00010-5

[19] La Rocca SA, Sandvik T. A short target real-time RT-PCR assay for detection of pestiviruses infecting cattle. J Virol Methods. 2009; 161; 122–127. 10.1016/j.jviromet.2009.06.005 19523981

[20] La Rocca SA, Cook R, Sandvik T, Strong R. Development of Strand-specific quantitative RT-PCR assay for bovine viral diarrhoea virus replication. 8th International Congress of Veterinary Virology. European Society for Veterinary Virology, Budapest, Hungary. 2009.

[21] Brownlie J, Clarke MC, Howard CJ, Pocock DH. Pathogenesis and epidemiology of bovine virus diarrhoea virus infection of cattle. Ann Rech Vet. 1987; 18; 157–166. 3619343

[22] Corapi WV, French TW, Dubovi EJ. Severe thrombocytopenia in young calves experimentally infected with noncytopathic bovine viral diarrhea virus. J Virol. 1989; 63; 3934–3943. 2548007 10.1128/jvi.63.9.3934-3943.1989 PMC250990

[23] Rebhun WC, French TW, Perdrizet JA, Dubovi EJ, Dill SG, Karcher LF. Thrombocytopenia associated with acute bovine virus diarrhea infection in cattle. J Vet Intern Med. 1989; 3; 42–46. 2926719 10.1111/j.1939-1676.1989.tb00327.x

[24] Howard CJ. Immunological responses to bovine virus diarrhoea virus infections. Rev Sci Tech. 1990; 9; 95–103. 1966728 10.20506/rst.9.1.488

[25] Larska M, Polak MP, Riitho V, Strong R, Belák S, Alenius S, et al Kinetics of single and dual infection of calves with an Asian atypical bovine pestivirus and a highly virulent strain of bovine viral diarrhoea virus 1. Comp Immunol Microbiol Infect Dis. 2012; 35; 381–390. 10.1016/j.cimid.2012.03.003 22480455

[26] Ridpath JF, Neill JD, Peterhans E. Impact of variation in acute virulence of BVDV1 strains on design of better vaccine efficacy challenge models. Vaccine. 2007; 25; 8058–8066. 17942196 10.1016/j.vaccine.2007.09.014

[27] Polak MP, Zmudzinski JF. Experimental inoculation of calves with laboratory strains of bovine viral diarrhea virus. Comp Immunol Microbiol Infect Dis. 2000; 23; 141–151. 10855660 10.1016/s0147-9571(99)00060-0

[28] Collins ME, Heaney J, Thomas CJ, Brownlie J. Infectivity of pestivirus following persistence of acute infection. Vet Microbiol. 2009; 138; 289–296. 10.1016/j.vetmic.2009.04.022 19443139

[29] Raya AI, Gomez-Villamandos JC, Sanchez-Cordon PJ, Bautista MJ. Virus Distribution and Role of Thymic Macrophages During Experimental Infection With Noncytopathogenic Bovine Viral Diarrhea Virus Type 1. Vet Pathol. 2012; 49; 811–818. 10.1177/0300985811414031 21768605

[30] Hamers C, Couvreur B, Dehan P, Letellier C, Fischer L, Brun AJ, et al Assessment of the clinical and virological protection provided by a commercial inactivated bovine viral diarrhoea virus genotype 1 vaccine against a BVDV genotype 2 challenge. Vet Rec. 2003; 153; 236–240. 13677324 10.1136/vr.153.8.236

[31] Nuttall PA. Growth characteristics of two strains of bovine virus diarrhoea virus. Arch Virol. 1980; 66; 365–369. 6255903 10.1007/BF01320633

[32] Castrucci G, Cilli V, Gagliardi G. Bovine virus diarrhea in Italy. I. Isolation and characterization of the virus. Arch Gesamte Virusforsch. 1968; 24; 48–64. 4971726 10.1007/BF01242901

[33] Odeon AC, Kelling CL, Marshall DJ, Estela ES, Dubovi EJ, Donis RO. Experimental infection of calves with bovine viral diarrhea virus genotype II (NY-93). J Vet Diagn Invest. 1999; 11; 221–228. 10353352 10.1177/104063879901100303

[34] Castrucci G, Osburn BI, Ferrari M, Traldi V. An experimental contribution to the study of the pathogenesis of bovine viral diarrhea virus infection. Comp Immunol Microbiol Infect Dis. 1992; 15; 163–169. 1325331 10.1016/0147-9571(92)90089-a

[35] Grooms DL, Brock KV, Ward LA. Detection of bovine viral diarrhea virus in the ovaries of cattle acutely infected with bovine viral diarrhea virus. J Vet Diagn Invest. 1998; 10; 125–129. 9576337 10.1177/104063879801000201

[36] Sandvik T, Drew T, Bensaude E, Davis L, Turner J, Brownlie J, et al. Acute BVD in calves—Effect of viral dose and immunosuppression on clinical signs of disease, transmission to susceptiable animals and virus persistence in tissues, 5th International Congress of the European Society for Veterinary Virology, Italy, 2000; pp. 401–402.

[37] Gogorza LM, Moran PE, Larghi JL, Segui R, Lissarrague C, Saracco M, et al Detection of bovine viral diarrhea virus (BVDV) in seropositive cattle. Prev Vet Med. 2005; 72; 49–54; discussion 215–219. 16253360 10.1016/j.prevetmed.2005.07.015

[38] Givens MD, Heath AM, Brock KV, Brodersen BW, Carson RL, Stringfellow DA. Detection of bovine viral diarrhea virus in semen obtained after inoculation of seronegative postpubertal bulls. Am J Vet Res. 2003; 64; 428–434. 12693532 10.2460/ajvr.2003.64.428

