# Supplementary figures and images for: Viral Dose and Immunosuppression Modulate the Progression of Acute BVDV-1 Infection in Calves: Evidence of Long Term Persistence after Intra-Nasal Infection
Source: PLoS One. 2015 May 8;10(5):e0124689. doi: 10.1371/journal.pone.0124689 (PMC4425503; doi:10.1371/journal.pone.0124689)

## Slide 1
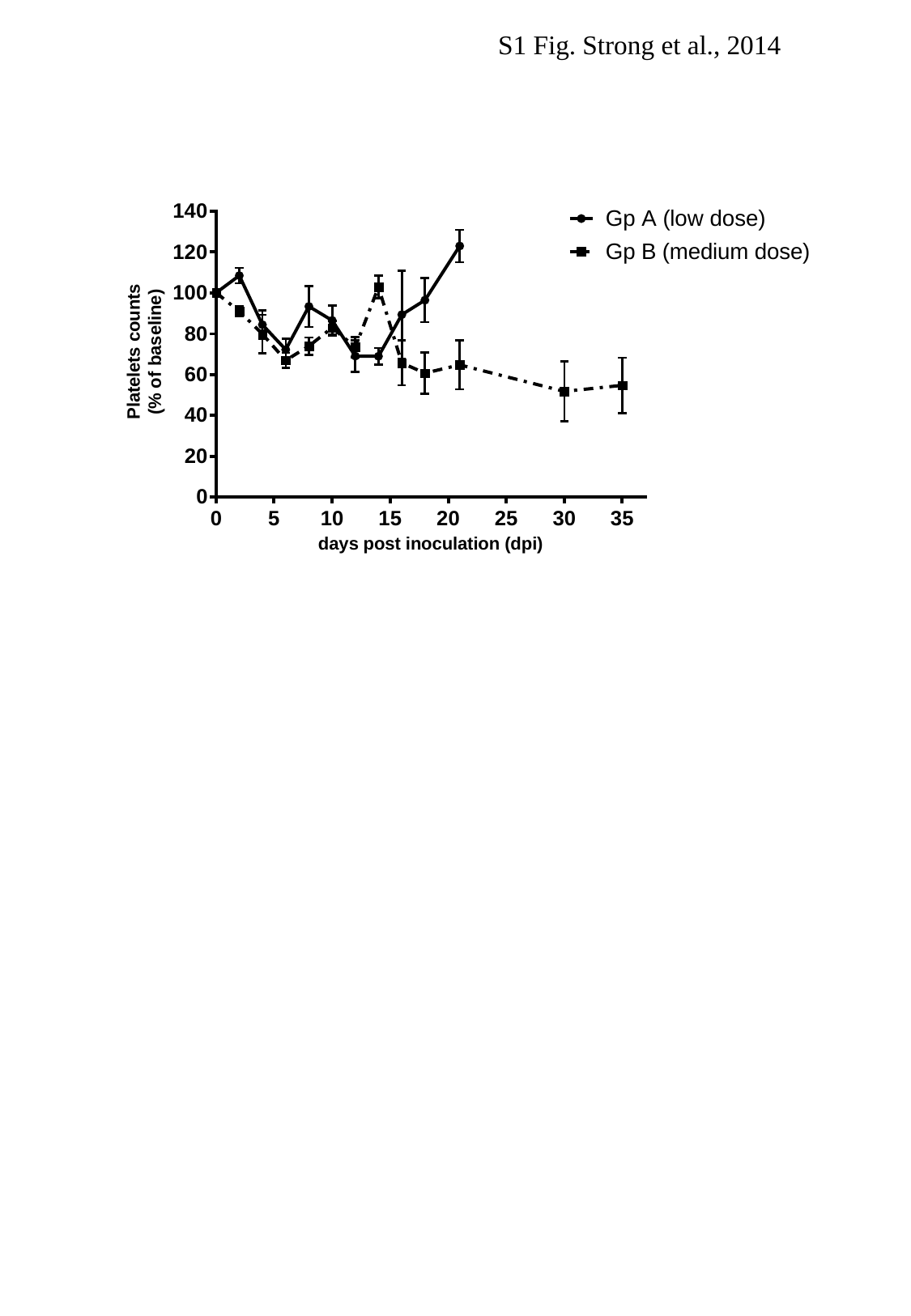

S1 Fig. Strong et al., 2014

Supplement: S1 Fig — Mean platelet counts ± SEM were plotted as a percentage of baseline counts in group A (low dose inoculum; only those animals that exhibited fever and/or positive by either RT-PCR or virus isolation were included e.g. 7 out of 12 calves) and group B (medium dose inoculum). WBC counts are shown for the acute phase of infection in group C (high dose inoculum). (PPT) [file pone.0124689.s001.ppt]

## Slide 1
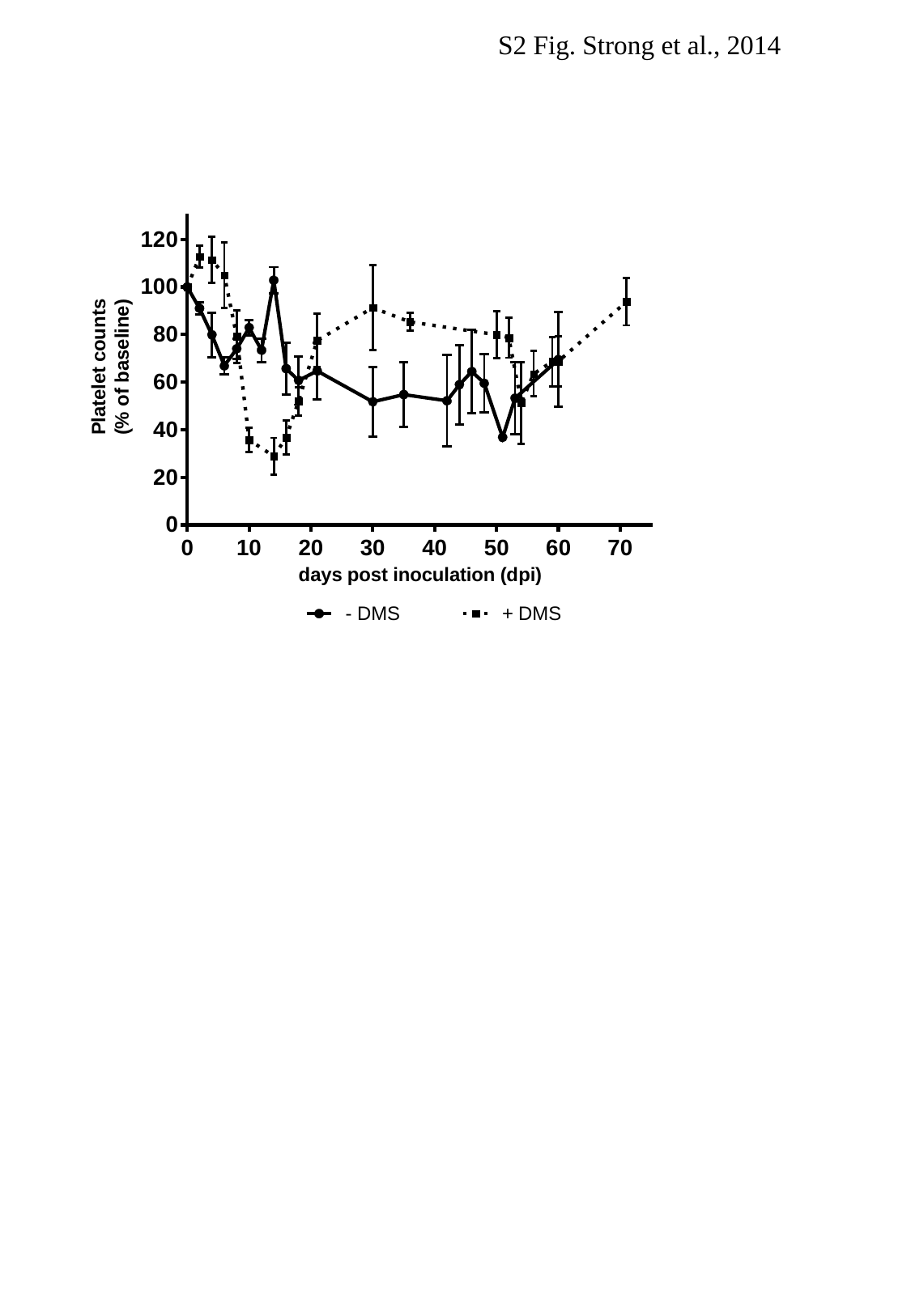

S2 Fig. Strong et al., 2014

Supplement: S2 Fig — Mean platelet counts ± SEM were plotted as percentages of baseline counts over the duration of the study, namely 60 dpi for group B (medium dose) and 71 dpi for group D (medium dose with DMS administered at time of inoculation). (PPT) [file pone.0124689.s002.ppt]

## Slide 1
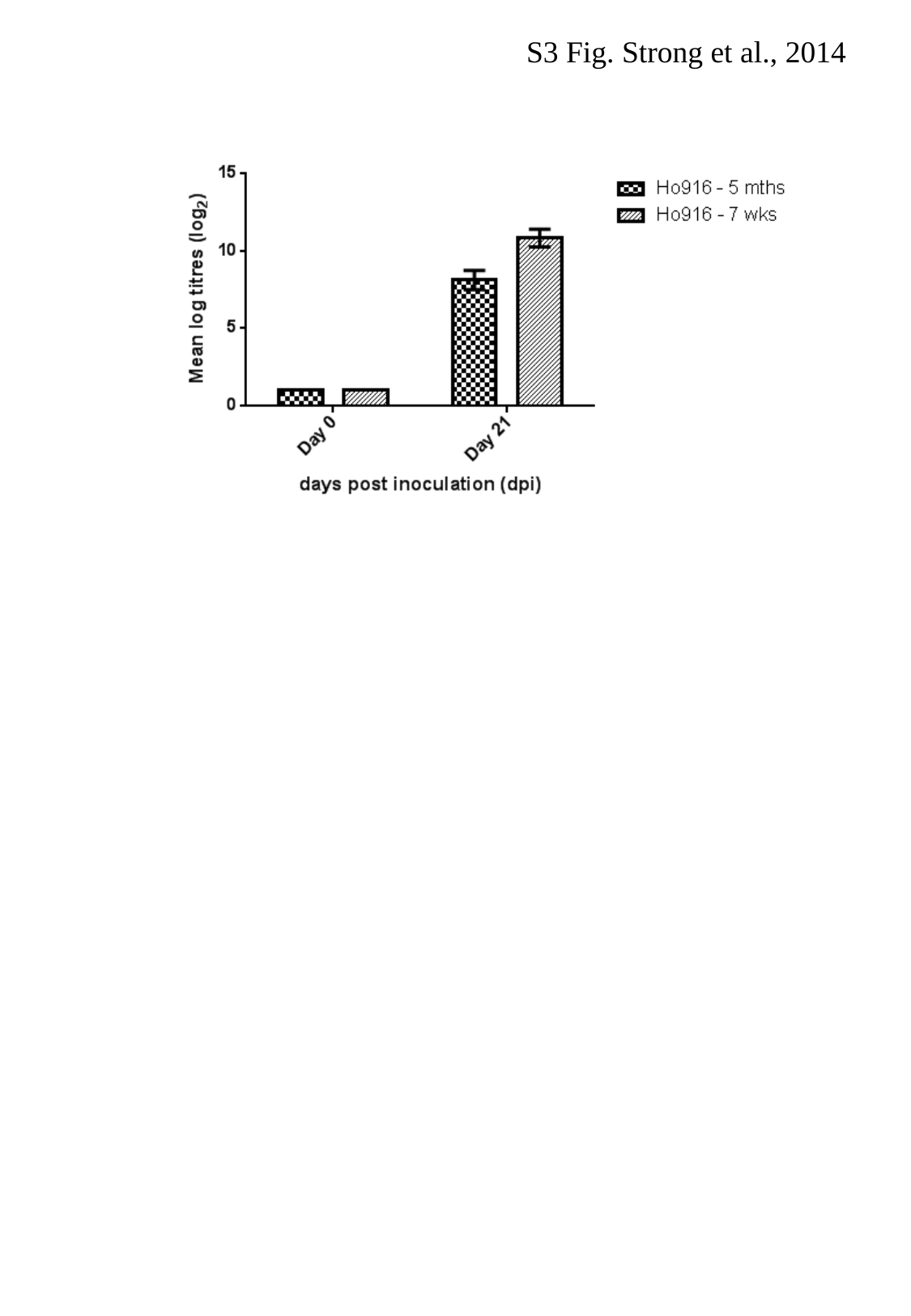

S3 Fig. Strong et al., 2014

Supplement: S3 Fig — Mean serum neutralising antibody titres (log2 transformed) ± SEM are plotted for group C (high dose inoculum) for the 7 weeks old and 5 month old animals. (PPT) [file pone.0124689.s003.ppt]
