# Supplementary material for: Viral Dose and Immunosuppression Modulate the Progression of Acute BVDV-1 Infection in Calves: Evidence of Long Term Persistence after Intra-Nasal Infection
Source: PLoS One. 2015 May 8;10(5):e0124689. doi: 10.1371/journal.pone.0124689 (PMC4425503; doi:10.1371/journal.pone.0124689)
Supplement: S1 Table — (DOC) [file pone.0124689.s004.doc]

| **Clinical Sign** | **Score** | | | |
| --- | --- | --- | --- | --- |
|  | Absent - 0 | Mild - 1 | Moderate – 2 | Severe – 3 |
| **Nasal Discharge** | No nasal discharge or small amount of clear secrete (is considered normal) | Intermittent watery-mucoid nasal discharge present | Persistent mucoid-mucopurulent nasal discharge | Severe nasal discharge (persistent purulent or haemorrhagic discharge) |
| **Ocular discharge** | No ocular discharge, eyes appear normal | Serous ocular discharge forming a streak down the cheek, eyes appear normal | Abundant serous ocular discharge wetting the side of the face and/or amount of mucopurulent discharge | Heavy tearing developing to thick purulent discharge, pain and/or swelling apparent in the eye, third eyelid and/or conjunctivitis |
| **Cough** | No coughing | Occasional spontaneous (dry) cough and/or unproductive induced* cough | Frequent spontaneous cough after movement and/or easily induced* productive cough | Frequent spontaneous cough at rest and/or induced* productive cough with prolonged episode |
| **Dyspnoea** | Normal respiratory effort and rate | Slightly increased respiratory effort and rate | Obvious abdominal breathing | Severe abdominal breathing  Mouth breathing  Two-staged expiration |
| **Depression** | Bright, alert, responsive  Normal behaviour  Staying with group | Reduced responsiveness  Otherwise normal behaviour | Depressed  Separates from group  Extended resting periods, reluctant to stand, lethargic | Unable to stand without assistance |
| **Diarrhoea** | Absent  Normal faeces | Pasty/soft faeces | Watery faeces | Bloody diarrhoea  Often associated with dehydration |
| **Inappetence** | Normal appetite | Slightly decreased appetite | Clearly decreased appetite | Anorexia |
| **Dehydration** | Absent  No dehydration | Slightly dehydrated | Skin tenting <2 seconds  Eyes slightly sunken | Skin tenting >3 seconds  Eyes sunken |

S1 Table. Clinical Scoring System

* Induced cough = cough that results from the application of moderate pressure on the animal’s trachea
